# Dose-Response Relationship between Cumulative Occupational Lead Exposure and the Associated Health Damages: A 20-Year Cohort Study of a Smelter in China

**DOI:** 10.3390/ijerph13030328

**Published:** 2016-03-16

**Authors:** Yue Wu, Jun-Ming Gu, Yun Huang, Yan-Ying Duan, Rui-Xue Huang, Jian-An Hu

**Affiliations:** 1 Department of Occupational and Environmental Health, School of Public Health, Central South University, 110# Xiang-ya Rd., Changsha 410078, Hunan, China; wuyue7802@csu.edu.cn (Y.W.); 13826391706m@sina.cn (J.-M.G.); lwhw2010@163.com (Y.H.); duanyy2010@126.com (Y.-Y.D.); arsenite@163.com (R.-X.H.); 2 Worker Hospital, Guangdong Shaoguan Smelter, Shaoguan 512024, Guangdong, China

**Keywords:** cumulative lead exposure, biological effects, lead poisoning, dose-response relationship, benchmark dose, occupational exposure limits

## Abstract

Long-term airborne lead exposure, even below official occupational limits, has been found to cause lead poisoning at higher frequencies than expected, which suggests that China’s existing occupational exposure limits should be reexamined. A retrospective cohort study was conducted on 1832 smelting workers from 1988 to 2008 in China. These were individuals who entered the plant and came into continuous contact with lead at work for longer than 3 months. The dose-response relationship between occupational cumulative lead exposure and lead poisoning, abnormal blood lead, urinary lead and erythrocyte zinc protoporphyrin (ZPP) were analyzed and the benchmark dose lower bound confidence limits (BMDLs) were calculated. Statistically significant positive correlations were found between cumulative lead dust and lead fumes exposures and workplace seniority, blood lead, urinary lead and ZPP values. A dose-response relationship was observed between cumulative lead dust or lead fumes exposure and lead poisoning (*p* < 0.01). The BMDLs of the cumulative occupational lead dust and fumes doses were 0.68 mg-year/m^3^ and 0.30 mg-year/m^3^ for lead poisoning, respectively. The BMDLs of workplace airborne lead concentrations associated with lead poisoning were 0.02 mg/m^3^ and 0.01 mg/m^3^ for occupational exposure lead dust and lead fume, respectively. In conclusion, BMDLs for airborne lead were lower than occupational exposure limits, suggesting that the occupational lead exposure limits need re-examination and adjustment. Occupational cumulative exposure limits (OCELs) should be established to better prevent occupational lead poisoning.

## 1. Introduction

Lead and its compounds are serious occupational and environmental pollutants. Acute lead poisoning usually presents as gastroenteritis in adults. Long-term lead exposure can cause encephalopathy, peripheral neuropathy, cognitive delays, anemia, and digestive and renal issues [1,2]. Chronic occupational lead poisoning is an issue in China, and unfortunately is also common in European countries and the U.S. [3,4]. Long-term lead exposure below occupational exposure limits (OELs) may cause a variety of sub-clinical problems, as well as chronic lead poisoning [5,6,7], suggesting that existing OELs may need amendment.

Studies of dose-response relationship between lead exposure and biological effects have focused primarily on the relationships between internal exposure dose (such as blood, urine, and bone lead) and effect biomarkers [8], which have great significance for biological lead exposure limits. Masci and colleagues reported that levels of lead exposure (airborne lead 0.0015–0.0240 mg/m^3^) affected hemoglobin synthesis [9]. Recent research has raised concerns regarding the toxicity of blood lead levels (BLLs) as low as 5 µg/dL [10]. Studies have reported that adults with BLLs of 25–60 µg/dL can exhibit nonspecific symptoms, including irritability, fatigue, headaches, sleep disturbance, decreased libido, and depressed mood [11]. The U.S. Department of Health and Human Services (DHHS) has recommended that BLLs be reduced to <25 µg/dL among all adults as a preventive health measure [12]. Internal exposure dose is an ideal indicator of the body’s lead burden because it takes various routes of entry into the body into account, which shows that more accurate and reasonable OELs are needed. Currently, biomarkers and lead dose-response relationships are not considered during the modification of these proposed OELs, because of contradictory and inconclusive results obtained from assessing the potential relationship between airborne lead exposure (external dose), individual doses (internal dose), and resultant biological effects [13,14]. The relationships between the external dose of lead and lead poisoning, and the related diagnostic biomarkers, remain unclear.

The benchmark dose (BMD) method is a novel technique that has been used to find toxicological reference doses (RfD) [15]. It has considerable scientific significance and a mathematical perspective useful to the analysis of dose-response effects. The method can be used on both quantal data, in which only the presence or absence of an effect is recorded, and on continuous data. There are also many reference dose models available for use with this method. A best-fitted model can be selected for the study of each dose-response relationship to produce results closely consistent with the original data. Many toxicologists have used the BMD method to study dose-response relationships in *in vivo* experiments and human biological exposure limits [16,17]. Some investigators have used BMD to analyze the dose-response toxicity data of occupational toxic agents, such as lead and cadmium [18,19].

Given that lead accumulates in the body (T_1/2_ = 5–10 years, accumulation in the tibia up to 48 years) health assessments and dose considerations should take the cumulative dose into account, especially in cases of long-term, low-dose exposure [20]. Herein, a cohort of lead smelting workers were retrospectively analyzed to determine levels of workplace lead exposure and their health status. Benchmark Dose Software (BMDS), developed by the U.S. Environmental Protection Agency (EPA), was used to analyze the dose-response relationship between occupational cumulative lead exposure and lead poisoning and biomarkers. This study provides references that can be used to adjust occupational exposure limits for airborne lead (dust and fumes) and supports the use of lead biomarkers for measuring occupational lead exposure.

## 2. Methods

### 2.1. Research Subjects

The smelter plant in this study is situated in southern China. The number of workers is relatively stable, and preventive work goes on regularly. Historical data regarding airborne lead concentration and employee medical reports were complete. A retrospective cohort study was conducted with smelting workers (*N* = 1832, 1654 men, 178 women) whose lead exposure duration was above 3 months from 1 January 1988 to 31 December 2008. The study excluded 51 workers with previous histories of occupational lead exposure and those who transferred to other units or moved between positions exposed to lead fumes and lead dust posts because no continuous data could be generated. Another 36 workers were lost to follow-up, resulting in a cohort of 1745 workers (1611 men, 134 women) with a mean age of 30.5 years (20–58 years) and a mean work seniority of 9.0 years (1–32.9 years). All subjects gave their informed consent for inclusion before participating in the study, which was conducted in accordance with the Declaration of Helsinki. The protocol was approved by the Ethics Committee of the Institute of Clinical Pharmacology (CTXY-080007-2).

### 2.2. Data Collection

Employee medical reports and airborne lead dust and lead fumes concentrations at the worksite were collected from the occupational healthcare unit. Airborne lead dust/fume were measured using stationary sampling at the worksite with MP-2 type samplers (Wuhan Analytical Instrument Company, Wuhan, China).The sampling head was held at breathing zone height (about 1.5 m above the ground) at representative worksite locations. The working hours of employees at different work sites were recorded for 8 h per day. The lead concentration was monitored quarterly.

### 2.3. Health Care and Monitoring

The workers’ health was assessed performed once a year according to the uniformly formulated Guideline of Occupational Health Surveillance (GBZ 188-2007), enacted by the Ministry of Health of the People’s Republic of China. Data collected included occupational exposure histories and subjective signs and symptoms of lead exposure. Urine was assessed for urinary coproporphyrin (CP) and blood was assessed for erythrocyte zinc protoporphyrin (ZPP). The lead poisoning diagnosis was performed by an occupational disease diagnosis group (more than three physicians with occupational qualifications) in accordance with the National Diagnostic Criteria for the Occupational Chronic Lead Poisoning (GBZ 37-2002) enacted by Ministry of Health of the People’s Republic of China (Table 1). Lead workers were diagnosed and divided into normal, observed object, and mild, moderate, and severe lead poisoning based on diagnostic criteria.

### 2.4. Laboratory Examinations

Here, 2 mL venous blood samples from the examined workers were collected in a lead-free EDTA tube; a first morning urine sample was also collected in a 100 mL lead-free polyethylene plastic bottles. Environmental lead content in air, blood, and urine was measured using an Analyst^TM^ 800 atomic absorption spectrometer (PerkinElmer, Inc., Shelton, CT, USA) using methods established by Qiao [21]. The detection limit was 0.5 μg/mL for air lead, which corresponds to an airborne level of 5 × 10^−4^ mg/m^3^ when sample volume was 50 m^3^. The detection limits were 0.3 μg/L and 1 μg /L for blood lead and urine lead, respectively. Control samples at low, medium, and high levels (AVIV) were run after every 20 readings and two levels of calibration samples (AVIV; low, high) were run twice per year. The reproducibility was 5.0%. The correlation coefficient of the calibration curve of the blood lead and urine lead was over 99.9%. ZPP was measured with a 206D type hematofluorometer (Aviv Biomedical, Inc., Lakewood, NJ, USA). The detection limits for ZPP was 0.1 μg/gHb. An ultraviolet fluorescent analysis of CP was performed with fluorescent light observation (ether layer with pale red fluorescence = “+”; an ether layer with significant red fluorescence/dark red fluorescence = “++” or “+++”).

### 2.5. Calculation of Cumulative Exposure

The daily time-weighted average (TWA) lead concentrations for workers from different positions were calculated using the following formula: 8 h TWA = (C_1_T_1_ + C_2_T_2_ +…+ C_n_T_n_)/8. C_n_ indicates the airborne lead concentrations at different job positions. T_n_ indicates the hours spent at the corresponding job position. The cumulative exposure dose was calculated from these values based on Haber’s Law, which states that D (mg year) = ∑ (C_j_), where C_j_ refers to the TWA concentration of any job position on year j, j = 1 to n, n is total lead exposed years by the cutoff year. The cutoff year was defined as the year of initial diagnosis of lead poisoning, or observed object, or the date of the last physical examination during which the subject had been diagnosed as normal. If the duration of exposure was less than one year, the exposure during that particular year (mg/m^3^) was measured as the 8 h TWA of that particular year × working days in that particular year/253. 253 days = (365 days − 11 days) × 5 days/7 days. There are 11 full-day annual holidays in China. The annual TWA concentration for each position was calculated using on-site monitoring data from different positions and locations during the year. Thus, cumulative exposure was the best estimate. Lead monitoring data in China did not use TWA standards until 2002, with the passage of the Occupational Diseases Prevention and Control Act. Before that, the annual average concentration of air lead of on-site monitoring was used for calculation of cumulative exposure.

### 2.6. Grouping Method

Workflow characteristics in the plant and the lead fumes or lead dust used as a risk factor in specific types of work were used to confirm positions exposed to lead fumes or lead dust. Statistical analyses were performed based different cumulative doses of lead dust/fume exposure.

### 2.7. Data Processing and Analyses

Databases were analysed using the Statistical Package for Social Sciences version 11.5 software (SPSS Inc., Chicago, IL, USA). A chi-square (χ^2^) analysis was used to compare various ratios. A pairwise comparison and linear correlation analysis between cumulative lead exposure, work seniority (years of occupational lead exposure), blood and urinary lead and ZPP was performed. Correlation analyses for lead poisoning and CP *vs.* cumulative exposure and other indicators were performed using a rank-correlation analysis. Diagnostic results of lead poisoning are here presented as scores of 1, 2, and 3, where 1 represents normal diagnosis/no lead poisoning; 2 represents observed object; and 3 represents lead poisoning (including mild, moderate, and severe lead poisoning). For CP a diagnosis, “+” was presented as 1, “++” was presented as 2 and “+++” was presented as 3.

For BMD calculations, the SPSS software was used to calculate cumulative lead exposure from each research subject (mg-year/m^3^). Research subjects were then divided groups according to cumulative lead exposure dose and the statistics for lead poisoning and ZPP. The BMDS (Version 2.2.1) from the U.S. EPA was used to calculate BMDs and BMDLs (the lower confidence limit of benchmark dose) for cumulative lead exposure, χ^2^ and *p*-value of corresponding Pearson’s chi-square tests for lead poisoning and ZPP, and dose-response relationship curves were drawn. In this study, several steps were included in the BMD/BMDL calculation [22]: (1) Benchmark dose reaction (BMR) was chosen; (2) Relevant parameters of appropriate models were selected; (3) The fitting of models was tested. In this process, a rejection level (0.05) was chosen to test whether or not the model fit is appropriate [18,23]. The *p*-value for testing whether or not the fitted model adequately describes the data is given next to the fitted model likelihood, and we can reject or not reject a hypothesis according to the *p*-value given. Small *p*-values indicate that a value of the goodness-of-fit statistic at least this extreme is unlikely to have been achieved if the data were actually sampled from the model, and, consequently, the model is a poor fit to the data; (4) BMDs and BMDLs for the adequate models were estimated; (5) BMD/BMDL from the model with lowest Akaike's Information Criterion (AIC) was fitted at a significance level of above 0.05.

## 3. Results

### 3.1. Basic Occupational Health

Participants of this study were chiefly men (*N* = 1745; 1611 men and 134 women; 664 cases of lead fumes exposure and 1081 cases of lead dust exposure) with a mean age of 30.5 years (20–58 years) and a mean work seniority of 9.0 years (1.0–32.9 years) (Table 2). Employment positions based on workflow were sintering, crushing, drying, acid-making, material preparation, slag fuming, refining, electrolysis, reverberatory furnace, gold and silver refining, lead melting and airing. Eight h TWA airborne lead concentrations for categories of work at the smelter over recent 20 years are provided in the Supplementary Materials, Table S1.

### 3.2. Correlation among Cumulative Lead Exposure, Lead Poisoning, and Biomarkers

Positive correlations were observed for cumulative lead dust exposure for workers and biomarkers, specifically for work seniority, blood and urinary lead, ZPP values, and lead poisoning (see supplemental sections, Table S2). Blood and urinary lead markers were positively correlated. Blood and urinary lead measurements were correlated with work seniority, lead poisoning and ZPP. Stronger correlations were observed between blood lead and lead poisoning, and between blood lead and ZPP than between urinary lead and either lead poisoning or ZPP (see the Supplementary Materials, Table S2). Positive correlations were found between cumulative lead fumes exposure and work seniority, blood and urinary lead, ZPP values and lead poisoning (see the Supplementary Materials, Table S3). Again, blood and urinary lead were positively correlated with lead poisoning and ZPP. Blood lead was more strongly correlated with lead poisoning and ZPP than was urinary lead.

### 3.3. Dose-Response Relationships between Cumulative Lead Exposure, Lead Poisoning and Biomarker ZPP

The lead exposure subjects were divided into five groups according to cumulative lead dust exposure. Data indicated that lead poisoning and ZPP increased with increased cumulative lead dust exposure, and this was statistically significant (Table 3). 

Lead fumes workers were divided into four groups according to cumulative lead fumes exposure; the data indicated that lead poisoning, ZPP increased with increasing cumulative lead fumes exposure, which was statistically significant (Table 4).

### 3.4. BMD and BMDLs for Lead Dust/Fume Exposure in Lead Poisoning and Other Biomarkers

**Figure 1:**
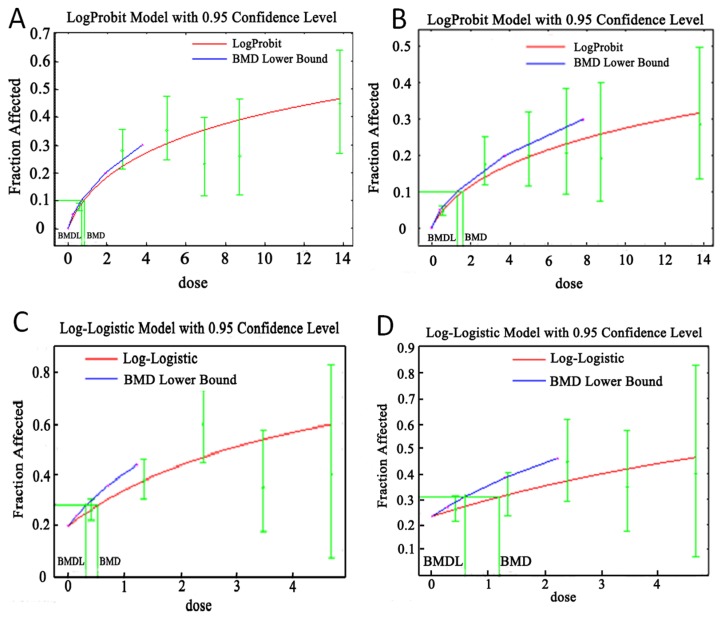
Dose-response relationships between cumulative lead dust/fume exposure and lead poisoning and abnormal ZPP. (**A**) BMD and BMDL of cumulative lead dust for lead poisoning; (**B**) BMD and BMDL of cumulative lead dust for abnormal ZPP; (**C**) BMD and BMDL of cumulative lead fumes for lead poisoning; (**D**) BMD and BMDL of cumulative lead fumes for abnormal ZPP, where the X-axis (*i.e.*, dose) represents cumulative lead dust/fume dose (mg·y/m^3^), and Y-axis (*i.e*., fraction affected) represented rates of lead poisoning (**A**,**C**) or abnormal ZPP (**B**,**D**). Abbreviations: ZPP, erythrocyte zinc protoporphyrin; BMD, benchmark dose; BMDL, benchmark dose lower bound.

The lead exposure subjects for BMD calculations were divided into five groups (0-, 2-, 4-, 6-, 8-, mg·y/m^3^) according to cumulative lead dust exposure, and four groups (0-, 1-, 2-, 3-, mg·y/m^3^) according to cumulative fume exposure. BMD and BMDLs for cumulative lead concentrations (mg/m^3^) based on dust and fume “working years” were assessed as well. For example, if an individual had worked for 30 years, BMD and BMDLs for cumulative concentration (mg/m^3^) were calculated as follows: BMD and BMDL cumulative dose (mg-year/m^3^)/30. Dose-response relationships between cumulative lead dust/fume exposure and lead poisoning, and ZPP were analysed using BMDS (Version 2.2.1) for both types of workers. BMD and BMDLs for airborne lead dust/fume concentrations were calculated from the model with lowest AIC among the models that adequately fit the data of BMR (an extra risk of 10%). Here, *p*-values above a significance level of 0.05 indicated that the models were adequately fit the data [18,23]. Results showed that Log-probit was representative for the lead dust, corresponding AIC (*p*-value) of which was 797.7 (0.076), 450.2 (0.665) for lead poisoning and ZPP (Table 5, Figure 1A,B). Log-logistic was representative for the lead fume, corresponding AIC (*p*-value) of which was 649.63 (0.073), 323.17 (0.383) for lead poisoning and ZPP (Table 5, Figure 1C,D). The BMDLs of the cumulative occupational lead dust and fumes doses were 0.68 mg-year/m^3^ and 0.30 mg-year/m^3^ for lead poisoning, respectively. The BMDLs of workplace airborne lead dust and lead fumes concentrations associated with lead poisoning were 0.02 mg/m^3^ and 0.01 mg/m^3^, respectively.

## 4. Discussion

Chronic lead poisoning is a frequent occupational disease that deserves attention [24]. Various strategies are currently used to measure dose-response relationships for lead exposure in order to measure environmental exposure (external doses) and internal doses (including blood, bone, hair, breast milk, saliva, fingernail, and urinary lead values). Internal dose indicators such as blood and urinary lead values can be used to quantify the amount of lead in the body and are routinely used to estimate occupational exposure because they are closely correlated with lead toxicity. Thus, the assessment of such biomarkers can be used to help improve the industry’s lead exposure standards and the diagnostic criteria related to occupational chronic lead poisoning [16,25]. The current work here indicates that these biomarkers can be used to estimate occupational airborne lead dust and lead fumes exposure and predict lead toxicity. Specifically, blood lead was found to be a more suitable internal dose indicator than urinary lead.

Internal dose indicators are used to detect lead that has already entered the body. This may, to a certain extent, reflect biological toxic effects. They can therefore be considered suitable for preclinical prevention measures (second level prevention). Occupational lead exposure occurs chiefly through breathing in lead particles; there are significant correlations between worksite airborne lead exposure and blood and urinary lead in workers [26,27]. Limiting occupational exposure to airborne lead is more important than second level prevention. External dose is expressed by airborne concentrations in the production environment, work seniority, or cumulative exposure. Ravichandan’s group [26] divided workers based on different lead concentrations in the workplace and measured blood lead, finding a correlation between higher airborne lead concentrations and blood lead. Variations in exposure times between different workers in different workplaces is often determined by seniority, so this factor has been included in several studies [28]; and the general consensus is that work seniority is not predictive of occupational lead doses. Cumulative lead exposure is a better estimate of potential lead toxicity than airborne concentrations or work seniority [29]. The cumulative dose which combines the duration of exposure and the current airborne lead concentration can be used to calculate levels of lead exposure in an entire workforce. *In vivo* and epidemiological studies have also suggested a significant dose-response relationship between chronic lead toxicity and cumulative lead exposure [30]. Increasing concentrations of inhaled lead accumulate in target organs; over time, these effects also have a dose-response relationship, with a greater rate of organ injury being proportional to lead exposure. Increased lead exposure also increases the number of workers with lead poisoning.

The U.S. Occupational Safety and Health Administration (OSHA) accepts a cumulative blood lead index (equivalent to multiplying the average blood lead level by the number of years of exposure) of 1600 µg-years/dL is an acceptable cumulative dose in the United States, in which a worker is exposed at the current OSHA-permissible limit and permitted to have the maximum blood lead level (40 µg/dL) each year for a working lifetime (40 years) [31]. The occupational lead cumulative exposure limits are not yet available at the moment in China and need exploration. Liao and colleagues applied annual job/industry-specific estimates of lead fumes and lead dust exposure to estimate cumulative exposure to lead fumes and lead dust of two prospective cohorts in Shanghai, China [32]. They found cumulative lead exposure to be positively associated with meningioma risk in women only, particularly with above-median cumulative exposure. In current study, subjects showed relatively stable lead exposure and received complete and regular occupational airborne lead monitoring as well as regular healthcare. The collected data helped to accurately measure cumulative lead exposure and analyze dose-response relationship between cumulative lead exposure and lead poisoning and changes in biomarkers.

Blood and urinary lead are required to diagnose occupational lead poisoning in China. ZPP (or FEP), urinary delta-ALA and CP are also considered to be suitable effect markers [8]. Our results show statistical correlations between cumulative lead exposure and blood lead, urinary lead, ZPP values and lead poisoning. Jangid’s group reported a strong correlation between blood lead and ZPP (*r* = 0.669, *p* < 0.001) [33], and the current results were consistent with those findings. Significant positive correlations were observed between blood lead and ZPP and cumulative lead exposure. The degree of correlation between CP and cumulative lead exposure/blood lead was less pronounced than what was observed between ZPP and cumulative lead exposure/blood lead. In this study, blood lead and ZPP were found to be more suitable for diagnosing lead poisoning than other factors. These findings are consistent with other studies of the effects of lead exposure that used blood lead and ZPP as biomarkers [26]. Cumulative lead exposure is fit for studying dose-response relationships between lead exposure and biological effects.

BMD has been used as a toxicological reference dose method (RfD). Compared with traditional no-observed-adverse-effect level (NOAEL) techniques, BMD is performed through a statistical analysis of all experimental data using a suitable dose-response model [34]. For this reason, most toxicology studies use BMD for *in vivo* experiments to measure dose-response relationships and biological exposure limits [35]. BMD has already been used to study the dose-response relationship between internal lead dose and effect markers in occupational epidemiology [6,18]. Tian *et al.*, estimated the BMD and BMDL of blood lead to explore the biologic exposure limits for renal dysfunction caused by lead [18]. BMD was used for a dose-response analysis between external exposure and health effects in an occupational population and compelling data were found. It is here, speculates that applying BMD to population data is a scientific and reasonable approach to the study dose-response relationships.

The current work suggests that lead poisoning and ZPP increase with cumulative lead exposure and that there is a dose-response relationship (Table 3 and Table 4). BMD and BMDLs for airborne lead dust exposure for regulating lead poisoning and the biomarker ZPP, are given in Table 5. BMDLs for occupational lead dust and fumes were 0.02 mg/m^3^ and 0.01 mg/m^3^, respectively, both lower than China’s national occupational exposure limits for each. China’s occupational exposure limit (OEL) for lead and inorganic compounds of lead was established in 1979, based on the maximum allowable concentrations of 0.05 mg/m^3^ for lead dust and 0.03 mg/m^3^ for lead fumes [36]. The OELs remained at the same levels but were based on time-weighted averages concentration from 2002 onward, which is similar to the United States’ standards for lead. In this way the existing national health standards for lead exposure pose unacceptable risks to workers with respect to unexpected lead poisoning. Revision of current health standards regarding lead exposure would protect workers’ health.

The current work is meant to answer some of the key questions necessary to analysis of the dose-response relationship between occupational cumulative lead exposure and the associated health damage and it reveals that existing national OEL for lead need adjustment. Some of the possible limitations of this study may have affected the analysis. Many other studies have also demonstrated that behavioral risk factors (e.g., tobacco and alcohol consumption); individual-level indicators (e.g., age, gender, socioeconomic status); health conditions (e.g., diabetes, heart disease, hypertension) and personal protection equipment may make have substantial effects on overall lead uptake [37,38]. Treating such factors solely as potential confounders would produce erroneous inferences regarding our findings. Additionally, men represent over 80% of the workers in this cohort so the bias of selected sample to the results on gender or other factors need to be taken into account.

## 5. Conclusions

In conclusion, cumulative lead exposure dose and the incidence of lead poisoning showed a significant dose-response relationship. The BMDLs of the cumulative occupational lead dust and fume doses were 0.68 mg-year/m^3^ and 0.30 mg-year/m^3^ for lead poisoning, respectively. This suggests that if the workers’ total cumulative dose of lead exposure can be controlled under these values, the incidence of lead poisoning will then be controlled within expected values. Occupational cumulative exposure limits (OCELs) should be established, so when occupational environmental lead concentrations exceed occupational exposure limits (first line of defense), the cumulative exposure dose of workers is controlled below OCELs (as a second line of defense) by reducing the total exposure time of workers, so preventing lead poisoning. BMDLs for airborne lead were lower than occupational exposure limits, suggesting that the occupational lead exposure limits need re-examination and adjustment.

## Figures and Tables

**Table 1:** National diagnostic criteria for the occupational chronic lead poisoning in China.

Grades	Diagnostic Criteria
Observed object	Have a close history of lead exposure, no clinical manifestations of lead poisoning, have one of the following performance:
	(1) Urinary lead ≥ 70 μg/L or 100 μg/24 h
	(2) Blood lead ≥ 40 μg/dL
Mild poisoning	Blood lead ≥ 60 μg/dL or urine lead ≥ 120 μg/L, and having one of the following performances:
	(1) Urinary δ-aminolevulinic acid (ALA) ≥ 8 mg/L
	(2) Blood free erythrocyte protoporphyrin (FEP) ≥ 200 μg/dL
	(3) Erythrocyte zinc protoporphyrin (ZPP) ≥ 2.91 μmol/L
	(4) Abdominal pain, bloating, constipation and other symptoms
Moderate poisoning	On the basis of mild poisoning, with one of the following symptoms:
	(1) Abdominal cramps
	(2) Anemia
	(3) Mild peripheral neuropathy
Severe poisoning	With one of the following manifestations:
	(1) Lead paralysis
	(2) Toxic encephalopathy

**Table 2:** Characteristics of the studied cohort.

Characteristics	Lead Dust Exposure		Lead Fumes Exposure		Total
	Men	Women	Men	Women	
Number of subjects	629	35	982	99	1745
Age (years)	30 (20–51)	34 (22–49)	30 (20–58)	32 (20–52)	30.5 (20–58)
Work seniority (years)	7.9 (1.0–31.7)	10.9 (1.8–26.6)	8.7 (1.0–32.9)	10.2 (1.0–28.5)	9.0 (1.0–32.9)

**Table 3:** Correlation between cumulative lead dust exposure and lead poisoning and ZPP.

Cumulative Dose (mg·y/m^3^)	*N*	Lead Poisoning (*N*)	Abnormal ZPP (*N*)
0-	913	68	41
2-	79	22	14
4-	37	13	7
6-	25	6	6
8-	27	9	8
χ^2^		78.061	64.224
*p*-value		<0.001	<0.001
Linear trend			
χ^2^		56.319	58.191
*p*-value		<0.001	<0.001

Note: 60 μg/dL blood lead, 120 mg/L urinary lead and 0.291 mmol/dL ZPP are diagnostic values for lead poisoning in China, and results equal or greater than these values were judged to be abnormal.

**Table 4:** Correlation between cumulative lead fumes exposure and lead poisoning and ZPP.

Cumulative Dose (mg·y/m^3^)	*N*	Lead Poisoning (*N*)	Abnormal ZPP (*N*)
0-	441	114	83
1-	148	56	37
2-	47	28	17
3-	28	10	10
χ^2^		26.748	7.024
*p*-value		<0.001	>0.05
Linear trend			
χ^2^		17.249	5.284
*p*-value		<0.001	<0.05

Note: 60 mg/dL blood lead, 120 mg/L urinary lead and 0.291 mmol/dL ZPP are diagnostic values for lead poisoning in China and results equal or greater than these values were judged to be abnormal.

**Table 5:** BMD and BMLD of lead dust and fume concentrations in lead poisoning and abnormal ZPP.

Effect Indicator	Types of Airborne Lead	Model	BMD (mg·y/m^3^)	BMD (mg/m^3^)	BMDL (mg·y/m^3^)	BMDL (mg/m^3^)	χ^2^-Value	*p*-Value *
Lead poisoning	dust	Log-probit	0.80	0.03	0.68	0.02	8.46	0.076
	fume	Log-logistic	0.47	0.02	0.30	0.01	6.98	0.073
ZPP	dust	Log-probit	1.62	0.05	1.30	0.04	2.39	0.665
	fume	Log-logistic	0.65	0.02	0.44	0.02	3.06	0.383

Notes: * *p*-values were determined from the chi-square test, with the Pearson goodness-of-fit test, to quantify the degree of the difference between predicted dose response with the fitted model and the actual dose response under each circumstance; *p* > 0.05 indicates that the model is adequately fit the data; Abbreviations: ZPP, erythrocyte zinc protoporphyrin; CP: coproporphyrin; BMD, benchmark dose; BMDL, benchmark dose lower bound.

## Supplementary Materials

The following are available online at www.mdpi.com/1660-4601/13/3/328/s1, Table S1: Airborne lead concentrations in different occupational categories (mean ± SD, mg/m^3^), Table S2: Pairwise correlations between cumulative lead dust exposure, work seniority and biomarkers, Table S3: Pairwise correlation between cumulative lead fume exposure, work seniority and biomarkers.

## Abbreviations

The following abbreviations are used in this manuscript:

ALA	urinary δ-aminolevulinic acid
BMD	Benchmark dose
BMDLs	95% lower confidence limit of the benchmark dose
CP	coproporphyrin
FEP	free erythrocyte protoporphyrin
OCELs	occupational cumulative exposure limits
PC-TWA	permissible concentration-time weighted average
T_1/2_	half-life
TWA	time-weighted average
ZPP	erythrocyte zinc protoporphyrin
