# Supplementary material for: Dose-Response Relationship between Cumulative Occupational Lead Exposure and the Associated Health Damages: A 20-Year Cohort Study of a Smelter in China
Source: Int J Environ Res Public Health. 2016 Mar 16;13(3):328. doi: 10.3390/ijerph13030328 (PMC4808991; doi:10.3390/ijerph13030328)
Supplement: Supplementary file 1 [file ijerph-13-00328-s001.pdf]

Supplementary Materials: Dose-Response Relationship between Occupational Cumulative lead Exposure and the Associated Health Damages: A 20-Year Cohort Study of a Smelter in China

Yue Wu, Jun-Ming Gu, Yun Huang, Yan-Ying Duan, Rui-Xue Huang and Jian-An Hu

Table S1. Airborne lead concentrations in different occupational categories (mean ± SD, mg/m^3).

Year	Sintering	Crushing	Drying	Acid-Making	Material Preparation	Slag Fuming	Refining	Electrolysis	Reverberator Furnace	Gold and Silver Refining	Airing *	Melting *
1988	0.28 ± 0.03	0.40 ± 0.04	0.22 ± 0.02	0.04 ± 0.01	0.21 ± 0.03	0.16 ± 0.01	0.12 ± 0.02	0.10 ± 0.01	0.06 ± 0.01	0.09 ± 0.01	0.11 ± 0.02	0.10 ± 0.01
1989	0.26 ± 0.04	0.34 ± 0.03	0.24 ± 0.03	0.06 ± 0.01	0.22 ± 0.04	0.14 ± 0.02	0.12 ± 0.01	0.06 ± 0.01	0.06 ± 0.01	0.09 ± 0.01	0.12 ± 0.02	0.09 ± 0.01
1990	0.26 ± 0.03	0.46 ± 0.05	0.23 ± 0.04	0.06 ± 0.01	0.21 ± 004	0.09 ± 0.01	0.10 ± 0.02	0.08 ± 0.01	0.04 ± 0.01	0.08 ± 0.01	0.10 ± 0.02	0.11 ± 0.01
1991	0.27 ± 0.02	0.47 ± 0.04	0.24 ± 0.04	0.06 ± 0.01	0.21 ± 0.02	0.13 ± 0.02	0.08 ± 0.01	0.10 ± 0.02	0.06 ± 0.01	0.10 ± 0.01	0.11 ± 0.02	0.08 ± 0.01
1992	0.38 ± 0.04	0.49 ± 0.03	0.23 ± 0.03	0.06 ± 0.01	0.20 ± 0.01	0.10 ± 0.02	0.06 ± 0.01	0.08 ± 0.01	0.07 ± 0.01	0.10 ± 0.01	0.10 ± 0.02	0.09 ± 0.01
1993	0.27 ± 0.04	0.56 ± 0.05	0.22 ± 0.03	0.05 ± 0.01	0.17 ± 0.03	0.10 ± 0.02	0.10 ± 0.01	0.10 ± 0.02	0.08 ± 0.01	0.09 ± 0.02	0.13 ± 0.02	0.09 ± 0.01
1994	0.40 ± 0.04	0.35 ± 0.04	0.21 ± 0.04	0.05 ± 0.01	0.15 ± 0.01	0.11 ± 0.01	0.07 ± 0.01	0.09 ± 0.01	0.08 ± 0.02	0.05 ± 0.01	0.10 ± 0.02	0.10 ± 0.01
1995	0.36 ± 0.05	0.63 ± 0.04	0.22 ± 0.02	0.07 ± 0.01	0.15 ± 0.03	0.10 ± 0.01	0.07 ± 0.01	0.09 ± 0.01	0.09 ± 0.01	0.07 ± 0.01	0.09 ± 0.02	0.08 ± 0.01
1996	0.23 ± 0.04	0.66 ± 0.05	0.17 ± 0.02	0.04 ± 0.01	0.08 ± 0.01	0.03 ± 0.01	0.10 ± 0.02	0.04 ± 0.00	0.05 ± 0.01	0.08 ± 0.01	0.11 ± 0.02	0.09 ± 0.01
1997	0.24 ± 0.04	0.51 ± 0.03	0.15 ± 0.04	0.04 ± 0.01	0.10 ± 0.02	0.04 ± 0.02	0.10 ± 0.01	0.06 ± 0.00	0.07 ± 0.01	0.07 ± 0.01	0.12 ± 0.02	0.07 ± 0.01
1998	0.19 ± 0.03	0.57 ± 0.05	0.17 ± 0.03	0.04 ± 0.01	0.12 ± 0.01	0.03 ± 0.00	0.11 ± 0.02	0.09 ± 0.01	0.08 ± 0.01	0.08 ± 0.01	0.11 ± 0.02	0.09 ± 0.01
1999	0.23 ± 0.04	0.73 ± 0.05	0.15 ± 0.02	0.06 ± 0.01	0.09 ± 0.01	0.05 ± 0.01	0.06 ± 0.01	0.10 ± 0.02	0.10 ± 0.01	0.10 ± 0.02	0.12 ± 0.02	0.10 ± 0.01
2000	0.21 ± 0.04	0.69 ± 0.08	0.10 ± 0.01	0.06 ± 0.01	0.09 ± 0.01	0.05 ± 0.01	0.06 ± 0.01	0.06 ± 0.01	0.09 ± 0.01	0.10 ± 0.01	0.12 ± 0.01	0.11 ± 0.02
2001	0.24 ± 0.04	0.63 ± 0.06	0.11 ± 0.02	0.04 ± 0.01	0.09 ± 0.01	0.03 ± 0.01	0.05 ± 0.01	0.04 ± 0.01	0.06 ± 0.01	0.07 ± 0.01	0.12 ± 0.02	0.11 ± 0.02
2002	0.26 ± 0.03	0.22 ± 0.04	0.08 ± 0.01	0.05 ± 0.02	0.10 ± 0.01	0.03 ± 0.01	0.04 ± 0.01	0.04 ± 0.00	0.04 ± 0.01	0.05 ± 0.01	0.13 ± 0.02	0.08 ± 0.01
2003	0.15 ± 0.02	0.17 ± 0.03	0.09 ± 0.02	0.04 ± 0.02	0.08 ± 0.01	0.04 ± 0.01	0.04 ± 0.01	0.04 ± 0.01	0.03 ± 0.02	0.05 ± 0.01	0.10 ± 0.01	0.07 ± 0.01
2004	0.19 ± 0.02	0.18 ± 0.02	0.11 ± 0.03	0.03 ± 0.01	0.05 ± 0.01	0.04 ± 0.01	0.07 ± 0.01	0.09 ± 0.01	0.02 ± 0.01	0.08 ± 0.01	0.08 ± 0.02	0.07 ± 0.01
2005	0.17 ± 0.04	0.24 ± 0.03	0.13 ± 0.03	0.05 ± 0.01	0.06 ± 0.01	0.04 ± 0.01	0.04 ± 0.00	0.06 ± 0.01	0.04 ± 0.01	0.07 ± 0.01	0.10 ± 0.02	0.08 ± 0.01
2006	0.20 ± 0.03	0.18 ± 0.03	0.08 ± 0.02	0.04 ± 0.01	0.05 ± 0.01	0.04 ± 0.01	0.03 ± 0.00	0.05 ± 0.01	0.04 ± 0.01	0.03 ± 0.00	0.12 ± 0.01	0.06 ± 0.01
2007	0.17 ± 0.03	0.17 ± 0.03	0.12 ± 0.02	0.04 ± 0.01	0.06 ± 0.01	0.03 ± 0.00	0.03 ± 0.01	0.04 ± 0.01	0.04 ± 0.00	0.04 ± 0.01	0.08 ± 0.01	0.09 ± 0.01
2008	0.19 ± 0.02	0.18 ± 0.01	0.17 ± 0.02	0.05 ± 0.01	0.08 ± 0.01	0.03 ± 0.00	0.04 ± 0.01	0.05 ± 0.01	0.06 ± 0.01	0.04 ± 0.00	0.09 ± 0.01	0.06 ± 0.01

Notes: Numbers of samples in every working category were 24 to 36 per year; * Represents occupational category that produces lead fumes; the absence of an asterisk indicates the occupational category that produces lead dust.

Table S2. Pairwise correlations between cumulative lead dust exposure, work seniority and biomarkers.

Variables	Cumulative Dose	Work Seniority	Blood Lead	Urinary Lead	ZPP	CP	Lead Poisoning
Cumulative dose	1	0.324 **	0.627 **	0.518 **	0.427 **	0.111 **	0.603 **
Work seniority	0.324 **	1	0.270 **	0.317 **	0.185 **	0.066	0.312 **
Blood lead	0.627 **	0.270 **	1	0.588 **	0.455 **	0.224 **	0.712 **
Urinary lead	0.518 **	0.317 **	0.588 **	1	0.231 **	0.229 **	0.550 **
ZPP	0.427 **	0.185 **	0.455 **	0.231 **	1	0.297 **	0.347 **
CP	0.111 **	0.066	0.224 **	0.229 **	0.297 **	1	0.532 **
Lead poisoning	0.603 **	0.312 **	0.712 **	0.550 **	0.347 **	0.532 **	1

Note: ** represents $p < 0.01$.

Table S3. Pairwise correlation between cumulative lead fume exposure, work seniority and biomarkers.

Variables	Cumulative Dose	Work Seniority	Blood Lead	Urinary Lead	ZPP	CP	Lead Poisoning
Cumulative dose	1	0.576 **	0.662 **	0.504 **	0.401 **	0.076 **	0.634 **
Work seniority	0.576 **	1	0.223 **	0.294 **	0.192 **	0.108 **	0.325 **
Blood lead	0.662 **	0.223 **	1	0.712 **	0.542 **	0.212 **	0.745 **
Urinary lead	0.504 **	0.294 **	0.712 **	1	0.193 **	0.185 **	0.635 **
ZPP	0.401 **	0.192 **	0.542 **	0.193 **	1	0.435 **	0.539 **
CP	0.076	0.108	0.212 **	0.185 **	0.435 **	1	0.456 **
Lead poisoning	0.634 **	0.325 **	0.745 **	0.635 **	0.539 **	0.456 **	1

Note: ** represents $p < 0.01$.

© 2016 by the authors; licensee MDPI, Basel, Switzerland. This article is an open access article distributed under the terms and conditions of the Creative Commons by Attribution (CC-BY) license (http://creativecommons.org/licenses/by/4.0/).
